# Anxiolytic and antidepressant effects of astaxanthin: behavioral and mechanistic insights in a rat model

**DOI:** 10.3389/fphar.2026.1748522

**Published:** 2026-03-18

**Authors:** Mohammad Ranjbari, Sajad Fakhri, Fatemeh Abbaszadeh, Amir Kiani, Ehsan Mohammadi-Noori, Javier Echeverría

**Affiliations:** 1 Student Research Committee, Kermanshah University of Medical Sciences, Kermanshah, Iran; 2 Pharmaceutical Sciences Research Center, Health Institute, Kermanshah University of Medical Sciences, Kermanshah, Iran; 3 Neurobiology Research Center, Institute of Neuroscience and Cognition, Shahid Beheshti University of Medical Sciences, Tehran, Iran; 4 Regenerative Medicine Research Center, Health Technology Institute, Kermanshah University of Medical Sciences, Kermanshah, Iran; 5 Departamento de Ciencias del Ambiente, Facultad de Química y Biología, Universidad de Santiago de Chile, Santiago, Chile

**Keywords:** anxiety, astaxanthin, depression, GABAergic system, muscarinic acetylcholine receptors, opioid system

## Abstract

**Background:**

Astaxanthin (AST) is a potent carotenoid with antioxidant properties that has garnered attention for its potential neuropharmacological effects.

**Purpose:**

This study investigates the anxiolytic and antidepressant activities of AST in a rat model to elucidate its therapeutic potential for mood disorders.

**Material and Methods:**

Fifty-four male rats were divided into nine groups receiving normal saline, astaxanthin (AST, 5, 10, and 15 mg/kg), diazepam (DZP, 0.5 mg/kg), fluoxetine (FXT, 5 mg/kg), or combinations of AST (10 mg/kg) with receptor antagonists flumazenil (FLU, GABA-A antagonist), atropine (ATR, muscarinic antagonist), and naloxone (NAL, opioid antagonist) for 14 consecutive days. At the end of the study, behavioral assessments were conducted, including the open field, light-dark box, elevated plus maze, tail suspension, and forced swimming tests. Biochemical analyses were performed to evaluate serum catalase (CAT), glutathione (GSH), nitrite, and matrix metalloproteinase-2 (MMP-2) and MMP-9 activities.

**Results and Discussion:**

Astaxanthin, particularly at 10 mg/kg, significantly reduced anxiety- and depressive-like behaviors, comparable to DZP and FXT in four-week-old male Wistar rats. Pretreatment with FLU, ATR, or NAL partially reversed these effects, suggesting involvement of GABAergic, cholinergic, and opioid pathways. Furthermore, AST enhanced antioxidant defenses, evidenced by increased serum CAT and GSH levels and reduced nitrite concentrations. Gelatin zymography revealed that AST increased MMP-2 activity while slightly decreasing MMP-9 activity, a partial reversal by the aforementioned antagonists.

**Conclusion:**

The results suggest that AST could produce anxiolytic and antidepressant effects. Such effects are likely mediated through GABAergic, cholinergic, and opioid systems, as well as antioxidant and anti-inflammatory mechanisms. Future studies should focus on well-controlled clinical trials to evaluate the preclinical results.

## 1 Introduction

Anxiety and depression are among the most prevalent mental health disorders globally, often co-occurring and intensifying their impact on individuals’ quality of life. The interaction between these conditions can result in a cycle of worsening symptoms, posing considerable challenges (Kendrick and Pilling, 2012; Kircanski et al., 2017). The emergence of anxiety and depression is influenced by a complex interplay of genetic, environmental, and neurobiological factors (Martin et al., 2009; Dean and Keshavan, 2017). A significant aspect of this relationship is shaped by the GABAergic system, particularly through GABA-A receptors. GABA (γ-aminobutyric acid) is the brain’s primary inhibitory neurotransmitter, and its dysregulation has been closely associated with various mood disorders. Clinical studies have shown that individuals with major depressive disorder (MDD) often exhibit reduced levels of GABA, a finding that correlates with increased depressive symptoms and heightened anxiety. The GABA-A receptor, an ionotropic receptor that mediates rapid synaptic inhibition, is crucial for regulating emotional states and responses to stress (Cutler et al., 2023). GABA-A receptors are composed of diverse subunit combinations, which significantly affect their pharmacological properties and behavioral outcomes. Evidence suggests that alterations in GABA-A receptor signaling can exacerbate anxiety and depressive behaviors (Fogaça and Duman, 2019). Notably, genetic modifications that reduce the expression of specific GABA-A receptor subunits have been associated with increased anxiety and depressive-like behaviors in animal models (Kalueff and Nutt, 2007).

Moreover, muscarinic acetylcholine receptors (mAChRs) also interact with the GABAergic system. These receptors are crucial for regulating neurotransmitter release and can impact the function of GABAergic interneurons (Yigit, 2003). Dysregulation of mAChRs has been implicated in mood disorders, indicating their potential role in the pathophysiology of both anxiety and depression (Je Jeon et al., 2015). The interaction between cholinergic signaling and GABAergic transmission may be crucial for emotional regulation, as both systems are essential for maintaining homeostasis within neural circuits associated with stress and mood (Mineur et al., 2022).

The opioid system adds another layer of complexity to the relationship between anxiety and depression (Peciña et al., 2019). Opioid receptors can modulate the release of various neurotransmitters, including GABA activation of opioid receptors has been shown to elicit anxiolytic effects, potentially alleviating anxiety symptoms; however, this interaction may also influence depressive states depending on the context (Reeves et al., 2022). The relationship between opioids and GABA-A receptors highlights a promising avenue for therapeutic interventions aimed at addressing both anxiety and mood disorders. By targeting these interconnected systems, novel treatments may emerge that offer more effective relief for individuals struggling with anxiety and depression (Fakhri et al., 2018).

Pharmacological treatments for anxiety and depression primarily include selective serotonin reuptake inhibitors (SSRIs) and benzodiazepines. While these medications can be effective, SSRIs typically require several weeks to reach their full effect, which can be frustrating for patients seeking immediate relief (Dunlop and Davis, 2008). Common side effects include sleep disturbances, sexual dysfunction, anxiety, weight changes, xerostomia, dizziness, headache, and gastrointestinal distress. There is evidence suggesting that SSRIs may be linked to an increased risk of suicidality, particularly in younger populations (Hu et al., 2004; Lochmann and Richardson, 2018). On the other hand, benzodiazepines work by enhancing the effect of the neurotransmitter GABA, which has a calming effect on the brain. This class of medications is typically used for short-term relief of severe anxiety or panic attacks (Goldschen-Ohm, 2022). Although benzodiazepines can provide rapid relief, they are associated with risks such as dependence, tolerance, cognitive impairment, and withdrawal symptoms, which limit their long-term use (Fluyau et al., 2018).

Given the limitations of conventional treatments, natural compounds such as astaxanthin (AST) have emerged as promising therapeutic candidates. Astaxanthin is a carotenoid pigment found in certain algae and seafood (such as salmon and shrimp) and is known for its potent antioxidant properties. As a green microalga, *Haematococcus pluvialis* is the major source of AST. The rich AST content of microalgae is produced in stress conditions, including nitrogen deficiency, high temperature, and high salinity. AST is also found in yeast *Xanthophyllomyces dendrorhous*, plants, a few fungi, *Chlorella zofingiensis*, *Chlorococcum* sp, and the marine bacterium *Agrobacterium aurantiacum* (Fakhri et al., 2018). Additionally, AST’s ability to cross the blood-brain barrier enhances its bioavailability within the central nervous system, potentially increasing its therapeutic effectiveness (Fakhri et al., 2018). Research has suggested that AST may have neuroprotective effects, potentially reducing oxidative stress and inflammation in the brain, both of which are thought to contribute to the development of mood disorders (Peng et al., 2023). Research suggests that AST may increase brain-derived neurotrophic factor (BDNF) levels in the prefrontal cortex of rats fed a high-fat diet and treated with streptozotocin, thereby enhancing neuroplasticity and improving mood-related behaviors (Ke et al., 2020). Regarding ethnopharmacological aspects, there is strong evidence of the traditional consumption and use of aquatic resources by humans. Around 145 species of seaweed are eaten, on occasion, with substantial health benefits. Seaweeds comprise about 10,000 species of macroalgae that live in subtidal and intertidal zones (Buckley et al., 2023).

Given the roles of the GABAergic system, mAChRs, and the opioid system in the context of anxiety and depression, this study aims to investigate whether AST exerts its therapeutic effects through these neurobiological mechanisms.

## 2 Materials and methods

### 2.1 Chemicals

Atropine sulfate (ATR), fluoxetine (FXT), flumazenil (FLU), and astaxanthin (AST; ≥98% purity) were acquired from Merck (Darmstadt, Germany). Naloxone (NAL) was sourced from GL Pharma GmbH (Lannach, Austria), and diazepam (DZP) was obtained from Ciba-Geigy (Basel, Switzerland). All medications were dissolved in normal saline to the required concentrations, ensuring a final injection volume of 10 mL/kg.

### 2.2 Animals

A total of 45 four-week-old male Wistar rats, weighing 220–250 g, were kept in a controlled environment at 22 °C ± 2 °C, 65% ± 2% humidity, and a 12-h light (8:00 a.m. to 20:00 p.m.)/dark (20:00 p.m. to 8:00 a.m.) cycle, with free access to water and a standard diet (RoshdDaneh company, Alborz, Iran). The rats were kept in cages measuring 26 cm × 42 cm × 15 cm, with 4 rats per cage. All experimental procedures were conducted between 9:00 a.m. and 2:00 p.m. The drugs were administered through intraperitoneal (i.p.) injections. All behavioral tests were performed following the guidelines approved by the Animal Care and Use Committee at Kermanshah University (IR.KUMS.REC.1400.547).

### 2.3 Experimental design

Three different doses of AST, 5, 10, and 15 mg/kg, were evaluated against standard anxiety and depression treatments, FXT (5 mg/kg) and DZP (0.5 mg/kg). A single injection of drugs was daily administered. Upon completion of the tests, the 10 mg/kg dose was identified as the most effective in achieving the desired outcomes. To further investigate the potential mechanisms behind the anxiolytic and antidepressant effects of AST, several antagonists were administered, including 0.5 mg/kg of FLU (a GABA-A receptor antagonist), 0.5 mg/kg of ATR (a muscarinic receptor antagonist), and 0.2 mg/kg of NAL (an opioid receptor antagonist). These antagonists were administered 1 hour before the experiment, followed by a 10 mg/kg dose of AST 30 min before testing, which was found to be the most effective dosage. Normal saline (NS, 0.2 cc each rat) was used as the vehicle for all groups.

In total, 54 rats were divided into 9 experimental groups, treated for 14 consecutive days, and subjected to behavioral evaluations as follows. NS, FXT, DZP, AST 5 mg/kg, AST 10 mg/kg, AST 15 mg/kg, FLU + AST 10 mg/kg, NAL + AST 10 mg/kg, and ATR + AST 10 mg/kg. A 30-min interval was observed between behavioral tests to prevent any interference with the results. Our previously published studies used the logic of determining the dosage of AST, FLU, and NAL (Hashemi et al., 2024), and we also used appropriate references for choosing the dosage of FXT (Deng et al., 2022), DZP (Mehrhoff et al., 2023), and ATR (Modarresi et al., 2020).

### 2.4 Behavioral tests

#### 2.4.1 Anxiolytic-like behavior tests

##### 2.4.1.1 Open field test

The open field test was used to assess locomotion, anxiety, and exploration (Prut and Belzung, 2003). The experimental area (W 100 cm × D 100 cm × H 40 cm) was divided into 12 equal squares. Each rat was placed in the central square during the test and observed for 5 min. Key parameters measured included the number of crossings (the total squares entered with all four paws), rearing (standing on hind legs), and grooming behavior, which can indicate the animal’s emotional state. After each trial, the box was thoroughly cleaned with 70% ethanol.

##### 2.4.1.2 Light-dark box test

The light-dark box made of Plexiglas® was divided into two equal compartments (25 cm × 25 cm × 25 cm each): one illuminated (the light or white compartment) and the other kept dark (the dark compartment). A 10 cm × 10 cm opening allowed for the free movement of the rats between the two areas. Each rat was initially placed in the center of the compartment, facing the light, and the experiment lasted for 5 min. In this test, the primary behavioral metrics recorded were the number of crossings between the light and dark compartments and the time spent in each compartment. The number of crossings served as an indicator of exploratory behavior. At the same time, the time ratio reflected the rats’ anxiety levels—typically, anxious rats avoid the light compartment, preferring the safety of darkness (Kulesskaya and Voikar, 2014).

##### 2.4.1.3 Elevated plus maze

The elevated plus maze is a behavioral assessment tool designed in a plus-shaped structure elevated 50 cm above the ground. It features two enclosed arms (50 cm long, 10 cm wide, and 40 cm high) connected by a central platform (10 cm × 10 cm) and two open arms (50 cm long and 10 cm wide) positioned perpendicularly. During the experiment, a rat was placed at the end of one closed arm, and its behavior was monitored for 5 min. Key parameters recorded include the time spent in the open versus closed arms and the number of crossings into the open arms, which helps assess anxiety-related behaviors (Danduga and Kola, 2024).

#### 2.4.2 Depressive-like behavior tests

##### 2.4.2.1 Tail suspension test

This test was employed as a supportive test to assess the antidepressant effects of AST. The rats were suspended by their tails, and the duration of immobility was measured. The duration of immobility is interpreted as a sign of despair or depression (Zhai et al., 2024).

##### 2.4.2.2 Forced-swimming test

The forced-swimming test was used for assessing depression-like behavior in rats. In this test, a rat was placed in a glass cylinder (D 50 cm × H 40 cm) containing 30 cm of water maintained at 25 °C. The rat was subjected to a 5-min forced swim test following a 1-min acclimation period. The primary metric assessed in the test was the duration of immobility, which indicates behavioral despair and symptoms related to depression (Porsolt et al., 1977; Gencturk and Unal, 2024; Arabi et al., 2025).

### 2.5 Biochemical analysis

At the end of the study and on day 14, blood samples were obtained from the rats’ retro-orbital sinus. The samples were then centrifuged (Sigma-Aldrich, St. Louis, United States) to separate the serum, subsequently frozen, and stored for biochemical and zymography analysis within a few days.

#### 2.5.1 Catalase and glutathione assay

The catalase activity was measured using the Aebi method, which assesses catalase’s ability to decompose hydrogen peroxide (H_2_O_2_) (Aebi, 1984). In this assay, 20 μL of serum and 100 μL of 65 mM H_2_O_2_ were incubated at 25 °C for 4 min (Memmert, Schwabach, Germany). The reaction was stopped by adding 100 μL of 32.4 mM ammonium molybdate, and the remaining H_2_O_2_ was quantified by measuring the absorbance of the yellow molybdate complex at 405 nm using an ELISA reader (Bio-Tek, United States).

For the glutathione (GSH) assay, the Ellman method was used (Tipple and Rogers, 2012; Fakhri et al., 2022). This method relies on the reaction of GSH with 5,5′-dithiobis (2-nitrobenzoic acid (DTNB), producing a 5′-thio-2-nitrobenzoic acid (TNB) chromophore whose formation is proportional to the GSH level in the sample. A mixture of 60 μL serum, 100 μL of 2 mg/mL DTNB, and 50 μL phosphate buffer was incubated for 10 min at 37 °C. The absorbance of the TNB chromophore was then measured at 412 nm.

In both assays, the results were expressed as the percentage difference in absorbance between the sample groups and the sham group.

#### 2.5.2 Nitrite assay

Nitrite levels in serum were measured using the Griess method (Sun et al., 2003). Serum (100 µL) was mixed with 50 µL of sulfanilamide solution (5% HCl) and incubated in the dark for 5 min. Then, 50 µL of 0.1% naphthyl ethylene diamine dihydrochloride (NEDD) was added, followed by a 30-min incubation at 37 °C in darkness. Absorbance was read at 540 nm, and a sodium nitrite standard curve was prepared for quantification.

### 2.6 Gelatin zymography

Gelatin zymography was conducted to evaluate gelatinase activity in serum samples containing 100 μg of total protein. Samples were loaded onto sodium dodecyl sulfate-polyacrylamide gel electrophoresis (SDS-PAGE) gels copolymerized with 0.1% gelatin and electrophoresed at 150 V. The gels were then washed with a renaturation buffer containing 2.5% Triton X-100 to restore enzyme function. Afterward, the gels were incubated overnight at 37 °C in a buffer containing sodium azide, calcium chloride, and sodium chloride to facilitate gelatin degradation. Finally, gels were stained with Coomassie blue, destained, and clear bands representing gelatinolytic activity were quantified using ImageJ software (Ren et al., 2017).

### 2.7 Statistical analysis

The data were analyzed using GraphPad Prism software (Version 8.3.4) and are presented as mean ± standard error of the mean (SEM). The normality of the data distribution was assessed with the Shapiro-Wilk test. Statistical assessments were carried out using one-way analysis of variance (ANOVA), followed by Tukey’s *post hoc* tests to identify group differences. A *p*-value of less than 0.05 was considered statistically significant.

## 3 Results

### 3.1 Astaxanthin reduced anxiolytic-like behavior in rats

#### 3.1.1 Open field test

**FIGURE 1:**
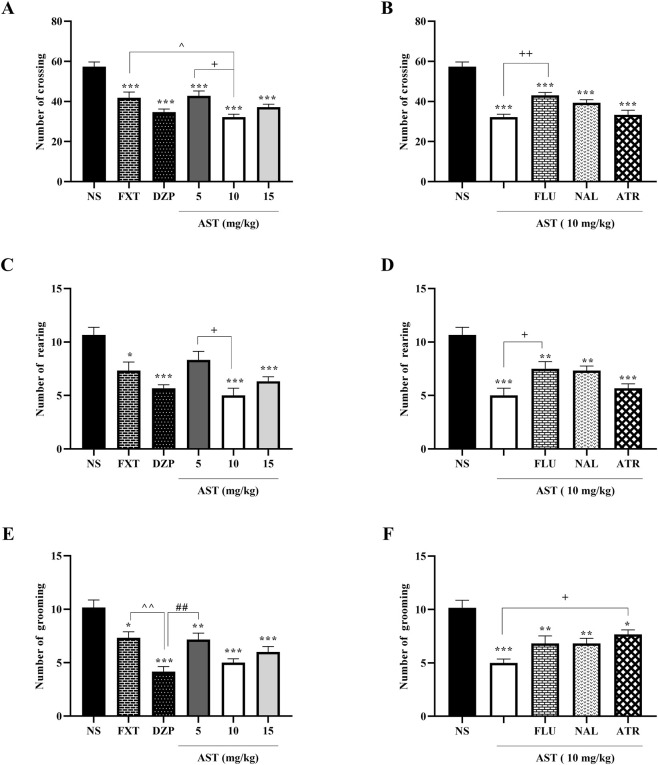
Effects of astaxanthin on exploratory behaviors in the open field test in rats. Fluoxetine (FXT, 5 mg/kg), diazepam (DZP, 0.5 mg/kg), and astaxanthin (AST, 5, 10, and 15 mg/kg) significantly reduced the number of crossings **(A)**, rearings **(C)**, and groomings **(E)** compared to normal saline (NS). Pretreatment of AST (10 mg/kg) with flumazenil (FLU, 0.5 mg/kg; GABA-A receptor antagonist), naloxone (NAL, 0.2 mg/kg; opioid receptor antagonist), or atropine (ATR, 0.5 mg/kg; muscarinic receptor antagonist) reversed the effects of AST on number of crossings **(B)**, rearings **(D)**, and groomings **(F)**. Data are presented as mean ± SEM, *n* = 6. Statistical analysis was performed using one-way ANOVA followed by Tukey’s *post hoc* test. ^*^ *p* < 0.05, ^**^ *p* < 0.01, ^***^ *p* < 0.001 vs. NS; ^∧^ *p* < 0.05, ^∧∧^ *p* < 0.01 vs. FXT; ^##^ *p* < 0.01 vs. DZP; ^+^ *p* < 0.05, ^++^ *p* < 0.01, vs. AST 10 mg/kg.

In this test, we examined key behavioral parameters -rearing, crossing, and grooming-in rats. One-way ANOVA revealed a significant effect of treatment on crossing behavior (F (5, 30) = 18.68, *p* < 0.001; Figure 1A). Post hoc Tukey’s test showed that rats treated with DZP (*p* < 0.001), FXT (*p* < 0.05), and AST (10 mg/kg; *p* < 0.001) significantly reduced crossing activity compared with the vehicle group (NS). Moreover, AST at 10 mg/kg significantly decreased crossing compared with AST 5 mg/kg (*p* < 0.05), indicating a dose-dependent effect.

Similarly, treatment had a significant effect on rearing behavior (F (5, 30) = 9.983, *p* < 0.001; Figure 1C). Post hoc analysis revealed that DZP (*p* < 0.001), FXT (*p* < 0.05), and AST (10 mg/kg; *p* < 0.001) significantly reduced rearing compared with the vehicle group, and AST 10 mg/kg also differed significantly from AST 5 mg/kg (*p* < 0.05).

For grooming behavior, one-way ANOVA showed a significant effect of treatment (F (5, 30) = 14.99, *p* < 0.001; Figure 1E). Post hoc Tukey’s test indicated that DZP (*p* < 0.001), FXT (*p* < 0.05), and AST (10 mg/kg; *p* < 0.001) significantly reduced grooming compared with the vehicle group.

To explore the underlying mechanisms of AST action, we employed FLU, NAL, and ATR as antagonists. One-way ANOVA revealed significant effects of antagonist pretreatment on AST-induced behavioral changes in crossing (F (4, 25) = 28.77, *p* < 0.001; Figure 1B), rearing (F (4, 25) = 13.54, *p* < 0.001; Figure 1D), and grooming (F (4, 25) = 11.52, *p* < 0.001; Figure 1F). Post hoc analysis showed that pretreatment with FLU, NAL, or ATR significantly reversed the effects of AST (*p* < 0.05–0.01), suggesting that the modulatory effects of AST are at least partly dependent on interactions with GABAergic, opioid, and cholinergic pathways.

#### 3.1.2 Light-dark box test

**FIGURE 2:**
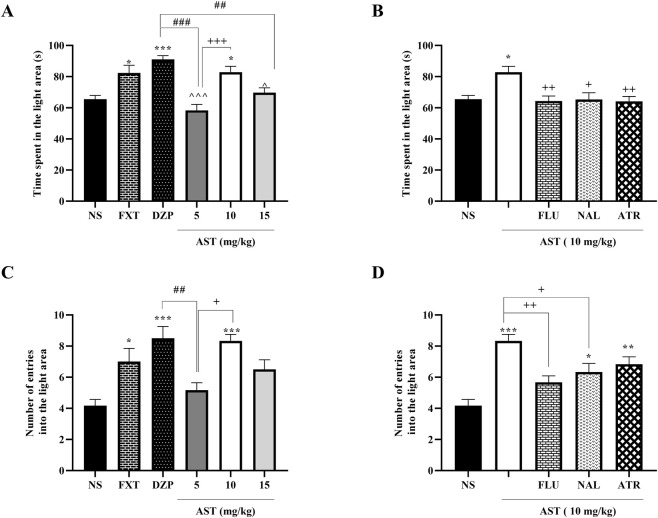
Effects of astaxanthin on behavior in the dark-light exploration test in rats. Fluoxetine (FXT, 0.5 mg/kg), diazepam (DZP, 1 mg/kg), or astaxanthin (AST, 10 and 15 mg/kg) significantly increased the time spent in the light area **(A)** and the number of entries into the light area **(C)** compared to normal saline (NS), indicative of anxiolytic-like activity. Pretreatment of AST (10 mg/kg) with flumazenil (FLU, 0.5 mg/kg; GABA-A receptor antagonist), naloxone (NAL, 0.2 mg/kg; opioid receptor antagonist), or atropine (ATR, 0.5 mg/kg; muscarinic receptor antagonist) reduced the anxiolytic-like effect of AST on time spent **(B)** and number of entries into the light area **(D)**. Data are presented as mean ± SEM, *n* = 6. Statistical analysis was performed using one-way ANOVA followed by Tukey’s *post hoc* test. ^*^ *p* < 0.05, ^**^ *p* < 0.01, ^***^ *p* < 0.001 vs. NS; ^∧^ *p* < 0.05, ^∧∧∧^ *p* < 0.001 vs. FXT; ^##^ *p* < 0.01, ^###^ *p* < 0.001 vs. DZP; ^+^ *p* < 0.05, ^++^ *p* < 0.01, ^+++^ *p* < 0.001 vs. AST 10 mg/kg.

Our results demonstrated significant treatment effects on anxiety-related behaviors in the dark–light exploration test. One-way ANOVA revealed a significant effect of treatment on the time spent in the light compartment (F (5, 30) = 12.28, *p* < 0.001; Figure 2A). Post hoc Tukey’s test showed that DZP (*p* < 0.001) and FXT (*p* < 0.05) significantly increased the time spent in the light area compared with the NS. Notably, AST at 10 mg/kg also significantly increased this parameter compared with NS (*p* < 0.05) and AST 5 mg/kg (*p* < 0.001), indicating a dose-dependent anxiolytic-like effect.

Similarly, one-way ANOVA indicated a significant treatment effect on the number of entries into the light compartment (F (5, 30) = 7.814, *p* < 0.001; Figure 2C). Post hoc Tukey’s analysis revealed that DZP (*p* < 0.001), FXT (*p* < 0.05), and AST at 10 mg/kg (*p* < 0.001) significantly increased the number of entries compared with the vehicle group. In addition, AST 10 mg/kg significantly differed from AST 5 mg/kg (*p* < 0.05), further supporting a dose-dependent effect.

To investigate the mechanisms underlying AST-induced anxiolytic-like effects, FLU, NAL, and ATR were used as pharmacological antagonists. One-way ANOVA showed a significant effect of antagonist pretreatment on the time spent in the light compartment (F (4, 25) = 5.61, *p* < 0.01; Figure 2B). Post hoc Tukey’s test demonstrated that pretreatment with FLU (*p* < 0.01), NAL (*p* < 0.05), and ATR (*p* < 0.01) significantly reversed the AST (10 mg/kg)-induced increase in time spent in the light area.

Moreover, antagonist pretreatment significantly affected the number of entries into the light area (F (4, 25) = 11.09, *p* < 0.001; Figure 2D). Post hoc analysis revealed that pretreatment with FLU (*p* < 0.01) and NAL (*p* < 0.05) markedly attenuated the AST-induced increase in light compartment entries.

#### 3.1.3 Elevated plus maze

**FIGURE 3:**
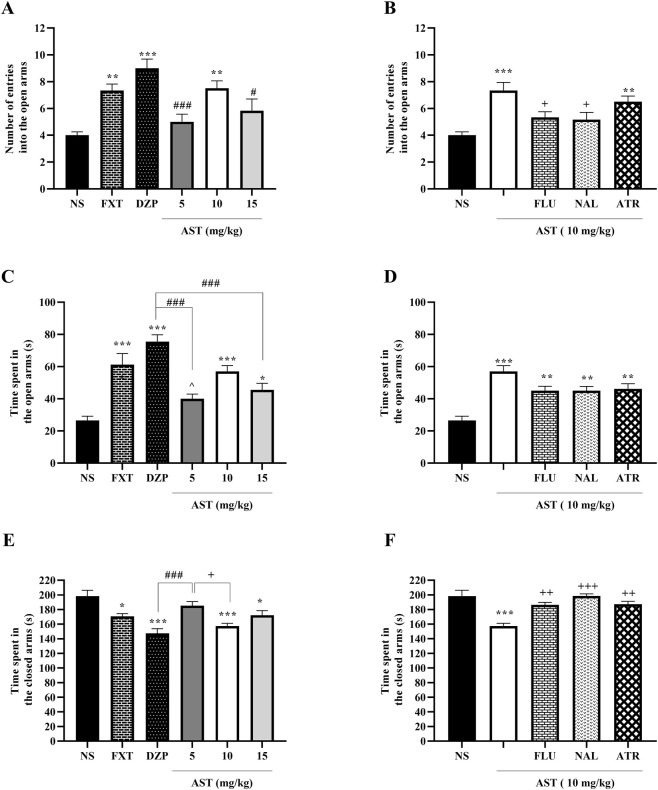
Effects of astaxanthin on exploratory behavior in the elevated plus maze test in rats. Fluoxetine (FXT, 0.5 mg/kg), diazepam (DZP, 1 mg/kg), or astaxanthin (AST, 10 mg/kg) significantly increased the number of entries into the open arms **(A)** and the time spent in the open arms **(C)** compared to normal saline (NS), while decreasing the time spent in the closed arms **(E)**. Pretreatment of AST (10 mg/kg) with flumazenil (FLU, 0.5 mg/kg; GABA-A receptor antagonist), naloxone (NAL, 0.2 mg/kg; opioid receptor antagonist), or atropine (ATR, 0.5 mg/kg; muscarinic receptor antagonist) reduced the anxiolytic-like effect on the number of entries into open arms **(B)**, time spent in open arms **(D)**, and time spent in closed arms **(F)**. Data are presented as mean ± SEM, *n* = 6. Statistical analysis was performed using one-way ANOVA followed by Tukey’s *post hoc* test. ^*^ *p* < 0.05, ^**^ *p* < 0.01, ^***^ *p* < 0.001 vs. NS; ^∧∧^ *p* < 0.01, ^∧∧∧^ *p* < 0.001 vs. FXT; ^##^ *p* < 0.01, ^###^ *p* < 0.001 vs. DZP; ^+^ *p* < 0.05, ^++^ *p* < 0.01, ^+++^ *p* < 0.001 vs. AST 10 mg/kg.

Our results revealed significant treatment-related alterations in anxiety-like behaviors in the elevated plus maze test. One-way ANOVA demonstrated a significant effect of treatment on the number of entries into the open arms (F (5, 30) = 9.25, *p* < 0.001; Figure 3A). Post hoc Tukey’s test indicated that DZP (*p* < 0.001), FXT (*p* < 0.01), and AST at 10 mg/kg (*p* < 0.01) significantly increased open-arm entries compared with the NS group.

Similarly, one-way ANOVA revealed a significant effect of treatment on time spent in the open arms (F (5, 30) = 15.71, *p* < 0.001; Figure 3C). Post hoc analysis showed that DZP (*p* < 0.001), FXT (*p* < 0.001), and AST at 10 mg/kg (*p* < 0.001) significantly increased open-arm time relative to the NS group.

Regarding the time spent in the closed arms, one-way ANOVA demonstrated a significant treatment effect (F (5, 30) = 9.97, *p* < 0.001; Figure 3E). Post hoc Tukey’s test revealed that DZP (*p* < 0.001), FXT (*p* < 0.05), and AST at 10 mg/kg (*p* < 0.001) significantly reduced closed-arm time compared with the vehicle group, while AST 10 mg/kg also differed significantly from AST 5 mg/kg (*p* < 0.05).

To explore the mechanisms contributing to AST-induced behavioral effects, animals were pretreated with pharmacological antagonists before AST (10 mg/kg) administration. One-way ANOVA showed a significant effect of antagonist pretreatment on the number of open-arm entries (F (4, 25) = 7.513, *p* < 0.001; Figure 3B). Post hoc Tukey’s analysis demonstrated that pretreatment with FLU (*p* < 0.05), and NAL (*p* < 0.05) significantly reversed the AST-induced increase in open-arm entries.

Likewise, antagonist pretreatment significantly affected time spent in the open arms, as revealed by a one-way ANOVA (F (4, 25) = 12.87, *p* < 0.001; Figure 3D). However, *post hoc* Tukey’s test indicated that pretreatment with FLU, NAL, and ATR produced a reduction in the AST (10 mg/kg)-induced increase in open-arm time, but this decrease did not reach statistical significance compared with the AST-treated group.

Finally, one-way ANOVA revealed a significant effect of antagonist pretreatment on the time spent in the closed arms (F (4, 25) = 12.27, *p* < 0.001; Figure 3F). Post hoc Tukey’s test showed that pretreatment with FLU (*p* < 0.01), NAL (*p* < 0.001), and ATR (*p* < 0.01) significantly increased closed-arm time compared with the AST (10 mg/kg)-treated group.

### 3.2 Astaxanthin reduced depressive-like behavior in rats

#### 3.2.1 Tail suspension test

**FIGURE 4:**
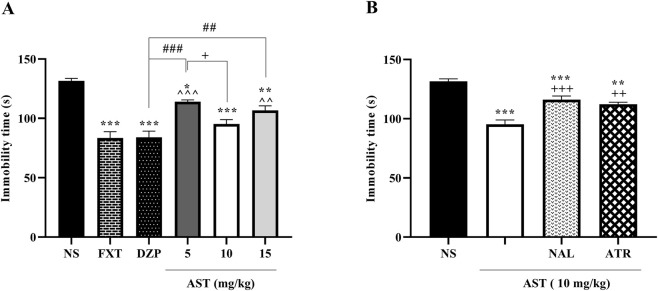
Effects of astaxanthin and receptor antagonists on the duration of immobility in the tail suspension test in rats: **(A)** Fluoxetine (FXT, 5 mg/kg), diazepam (DZP, 0.5 mg/kg), and astaxanthin (AST, 5, 10, and 15 mg/kg) significantly reduced immobility compared to normal saline (NS), indicative of antidepressant-like activity. **(B)** Pretreatment of AST (10 mg/kg) with naloxone (NAL, 0.2 mg/kg; opioid receptor antagonist) or atropine (ATR, 0.5 mg/kg; muscarinic receptor antagonist) markedly reversed the antidepressant effect of AST. Data are presented as mean ± SEM, *n* = 6. Statistical analysis was performed using one-way ANOVA followed by Tukey’s *post hoc* test. ^*^ *p* < 0.05, ^**^ *p* < 0.01, ^***^ *p* < 0.001 vs. NS; ^∧^ *p* < 0.05 vs. FXT; ^#^ *p* < 0.05, ^###^ *p* < 0.001 vs. DZP; ^+^ *p* < 0.05, ^++^ *p* < 0.01, ^+++^ *p* < 0.001 vs. AST 10 mg/kg.

To evaluate depressive-like behavior, immobility time was assessed in the tail suspension test. One-way ANOVA revealed a significant effect of treatment on immobility time (F (5, 30) = 22.85, *p* < 0.001; Figure 4A). Post hoc Tukey’s test showed that FXT (*p* < 0.001) and DZP (*p* < 0.001) significantly reduced immobility time compared with the NS group. Similarly, AST treatment produced a dose-dependent antidepressant-like effect, as AST at 10 mg/kg (*p* < 0.001) and 15 mg/kg (*p* < 0.01) significantly decreased immobility time relative to NS, while AST at 5 mg/kg induced a smaller but still significant reduction (*p* < 0.05) vs. NS and AST (10 mg/kg).

To further examine factors contributing to the effects of AST, pharmacological antagonists were administered prior to AST (10 mg/kg). One-way ANOVA demonstrated a significant effect of antagonist pretreatment on immobility time (F (3, 20) = 28.66, *p* < 0.001; Figure 4B). Post hoc Tukey’s analysis revealed that pretreatment with NAL significantly increased immobility time compared with the AST-treated group (*p* < 0.001). Likewise, ATR pretreatment also significantly attenuated the AST-induced reduction in immobility time (*p* < 0.01).

#### 3.2.2 Forced-swimming test

**FIGURE 5:**
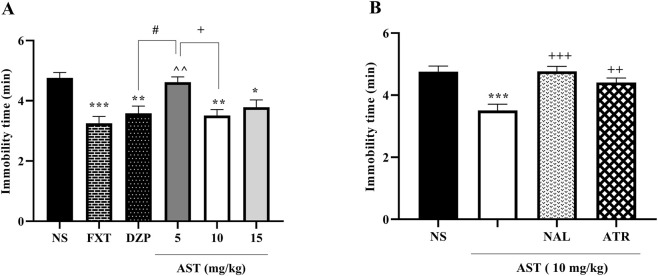
Effects of astaxanthin on immobility time in the forced swimming test in rats. **(A)** Fluoxetine (FXT, 5 mg/kg), diazepam (DZP, 0.5 mg/kg), and astaxanthin (AST, 10 and 15 mg/kg) significantly reduced immobility compared with normal saline (NS), indicating antidepressant-like effects. **(B)** Pretreatment of AST (10 mg/kg) with naloxone (NAL, 0.2 mg/kg, opioid receptor antagonist) and atropine (ATR, 0.5 mg/kg, muscarinic receptor antagonist) reversed the antidepressant effect of AST (10 mg/kg). Data are expressed as mean ± SEM, *n* = 6. Statistical analysis was performed using one-way ANOVA followed by Tukey’s *post hoc* test. ^*^ *p* < 0.05, ^**^ *p* < 0.01, ^***^ *p* < 0.001 vs. NS; ^∧∧^ *p* < 0.01 vs. FXT; ^#^ *p* < 0.05 vs. DZP; ^+^ *p* < 0.05, ^++^ *p* < 0.01, ^+++^ *p* < 0.001 vs. AST 10 mg/kg.

Our results revealed significant treatment-related alterations in depressive-like behavior as assessed by the forced-swimming test. One-way ANOVA demonstrated a significant effect of treatment on immobility time (F (5, 30) = 8.632, *p* < 0.001; Figure 5A). Post hoc Tukey’s test showed that the reference antidepressant drugs DZP (*p* < 0.001) and FXT (*p* < 0.001) significantly reduced immobility time compared with the NS group. Notably, AST administration at doses of 10 mg/kg (*p* < 0.01) and 15 mg/kg (*p* < 0.05) also resulted in a significant decrease in immobility time relative to NS, indicating an antidepressant-like effect. Moreover, AST at 10 mg/kg significantly differed from the lower dose of 5 mg/kg (*p* < 0.05), suggesting a dose-dependent response.

To further explore the underlying mechanisms, the involvement of opioid and muscarinic cholinergic receptors was examined. One-way ANOVA revealed a significant effect of pretreatment on immobility time (F (3, 20) = 11.77, *p* < 0.001; Figure 5B). Post hoc analysis demonstrated that pretreatment with NAL (*p* < 0.001), and ATR (*p* < 0.01), significantly reversed the antidepressant-like effect of AST (10 mg/kg), indicating that opioid and muscarinic cholinergic systems may contribute to the observed behavioral effects of AST.

### 3.3 Astaxanthin reduced oxidative and nitrosative stress

**FIGURE 6:**
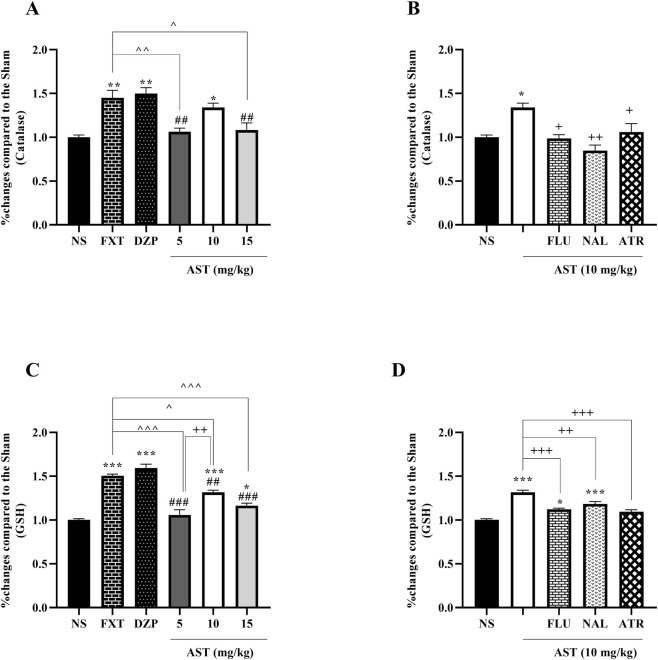
Effects of astaxanthin on antioxidant enzyme activity in the rats. Fluoxetine (FXT, 5 mg/kg), diazepam (DZP, 0.5 mg/kg), and astaxanthin (AST, 10 and 15 mg/kg) significantly increased catalase (CAT) **(A)** and glutathione (GSH) **(C)** levels compared to normal saline (NS). Pretreatment of AST (10 mg/kg) with flumazenil (FLU, 0.5 mg/kg; GABA-A receptor antagonist), naloxone (NAL, 0.2 mg/kg; opioid receptor antagonist), or atropine (ATR, 0.5 mg/kg; muscarinic receptor antagonist) reduced these antioxidant effects **(B,D)**. Data are presented as mean ± SEM, *n* = 3. Statistical analysis was performed using one-way ANOVA followed by Tukey’s *post hoc* test. ^*^ *p* < 0.05, ^**^ *p* < 0.01, ^***^ *p* < 0.001 vs. NS; ^∧^ *p* < 0.05, ^∧∧^ *p* < 0.01, ^∧∧∧^ *p* < 0.001 vs. FXT; ^##^ *p* < 0.01, ^###^ *p* < 0.001 vs. DZP; ^+^ *p* < 0.05, ^++^ *p* < 0.01, ^+++^ *p* < 0.001 vs. AST 10 mg/kg.

Our results demonstrated that AST markedly attenuated oxidative stress by enhancing endogenous antioxidant defenses. One-way ANOVA revealed a significant effect of treatments on catalase activity (F (5, 12) = 12.09, *p* < 0.001; Figure 6A). Post hoc Tukey’s test showed that the standard drugs FXT and DZP significantly increased serum catalase activity compared with the NS group (*p* < 0.01). Similarly, AST administration significantly elevated catalase levels, with the 10 mg/kg dose producing a significant increase compared with NS (*p* < 0.05) and exhibiting an effect comparable to that of FXT and DZP.

Likewise, one-way ANOVA demonstrated a significant effect of treatments on reduced glutathione (GSH) levels (F (5, 12) = 46.30, *p* < 0.001; Figure 6C). Post hoc analysis indicated that FXT and DZP markedly increased GSH levels relative to the NS group (*p* < 0.001). AST treatment also significantly enhanced GSH levels at all tested doses, with the 10 mg/kg dose exerting the most pronounced effect (*p* < 0.001 vs. NS), comparable to the antioxidant effects observed with FXT and DZP. Moreover, AST at 10 mg/kg significantly differed from the lower dose of 5 mg/kg (*p* < 0.01), suggesting a dose-dependent response.

To further elucidate the mechanisms underlying the antioxidant effects of AST, the involvement of GABAergic (benzodiazepine site–related), opioid, and muscarinic cholinergic pathways was examined using specific antagonists. One-way ANOVA revealed a significant effect of antagonist pretreatment on catalase activity (F (4, 10) = 9.17, *p* < 0.01; Figure 6B). Post hoc Tukey’s test demonstrated that pretreatment with FLU (*p* < 0.05), NAL (*p* < 0.01), or ATR (*p* < 0.05) significantly attenuated the AST-induced increase in catalase activity compared with AST treatment alone.

Consistently, one-way ANOVA showed a significant effect of antagonist pretreatment on GSH levels (F (4, 10) = 30.92, *p* < 0.001; Figure 6D). Post hoc analysis revealed that pretreatment with FLU (*p* < 0.001), NAL (*p* < 0.01), or ATR (*p* < 0.001) significantly reversed the AST-induced elevation of GSH levels compared with the AST-treated group.

**FIGURE 7:**
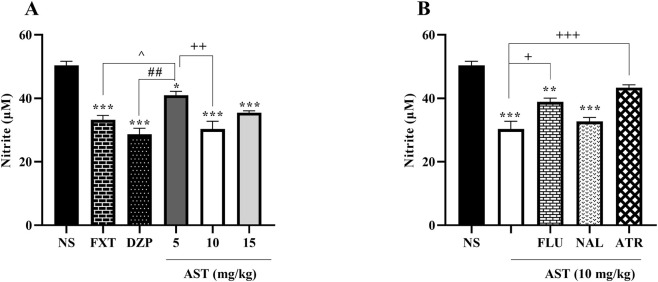
Effects of astaxanthin on nitrite levels in the rats. **(A)** Fluoxetine (FXT, 5 mg/kg), diazepam (DZP, 0.5 mg/kg), and astaxanthin (AST, 5, 10, and 15 mg/kg, respectively) significantly reduced nitrite levels compared with normal saline (NS). **(B)** Pretreatment of AST (10 mg/kg) with flumazenil (FLU, 0.5 mg/kg; GABA-A receptor antagonist), naloxone (NAL, 0.2 mg/kg; opioid receptor antagonist), or atropine (ATR, 0.5 mg/kg; muscarinic receptor antagonist) reversed the effect of AST (10 mg/kg) on nitrite levels. Data are presented as mean ± SEM, *n* = 3. Statistical analysis was performed using one-way ANOVA followed by Tukey’s *post hoc* test. ^*^ *p* < 0.05, ^**^ *p* < 0.01, ^***^ *p* < 0.001 vs. NS; ^^^ *p* < 0.05 vs. FXT; ^##^ *p* < 0.01 vs. DZP; ^+^ *p* < 0.05, ^++^ *p* < 0.01, ^+++^ *p* < 0.001 vs. AST 10 mg/kg.

Additionally, serum nitrite levels, as an indicator of nitrosative stress, were significantly altered following the different treatments. One-way ANOVA revealed a significant effect of treatment on nitrite concentrations (F (5, 12) = 25.57, *p* < 0.001; Figure 7A). Post hoc Tukey’s test showed that FXT and DZP significantly reduced serum nitrite levels compared with the NS control group (*p* < 0.001). Similarly, AST administration produced a significant reduction in nitrite concentrations, with the most pronounced effect observed at 10 and 15 mg/kg (*p* < 0.001 vs. NS). Moreover, AST at 10 mg/kg significantly differed from the lower dose of 5 mg/kg (*p* < 0.01), further supporting a dose-dependent protective effect against nitrosative stress.

To further investigate the pathways involved in AST-mediated modulation of nitrite levels, antagonist pretreatments were applied prior to AST (10 mg/kg). One-way ANOVA demonstrated a significant effect of antagonist pretreatment on serum nitrite concentrations (F (4, 10) = 29.12, *p* < 0.001; Figure 7B). Post hoc analysis indicated that pretreatment with FLU (*p* < 0.05) and ATR (*p* < 0.001) significantly reversed the AST-induced reduction in nitrite levels compared with the AST-treated group alone.

### 3.4 Astaxanthin modulated serum activity of inflammatory MMP-2 and MMP-9

**FIGURE 8:**
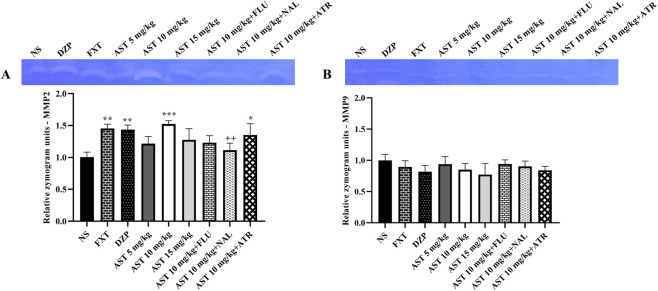
Effects of astaxanthin on matrix metalloproteinase (MMP) activity in rats. **(A)** Fluoxetine (FXT, 5 mg/kg), diazepam (DZP, 0.5 mg/kg), and astaxanthin (AST, 10 mg/kg) significantly increased MMP2 activity compared with normal saline (NS). Pretreatment of AST (10 mg/kg) with flumazenil (FLU, 0.5 mg/kg; GABA-A receptor antagonist), naloxone (NAL, 0.2 mg/kg; opioid receptor antagonist), or atropine (ATR, 0.5 mg/kg; muscarinic receptor antagonist) reduced the effect of AST (10 mg/kg). **(B)** Representative zymograms and quantitative analysis of MMP-9 activity under the same treatment conditions, showing no significant changes. Data are presented as mean ± SEM, *n* = 3. Statistical analysis was performed using one-way ANOVA followed by Tukey’s *post hoc* test. ^*^ *p* < 0.05, ^**^ *p* < 0.01, ^***^ *p* < 0.001 vs. NS; ^++^ *p* < 0.01 vs. AST 10 mg/kg.

Zymography analysis revealed significant treatment-related alterations in MMP-2 activity. One-way ANOVA demonstrated a significant effect of different treatments on MMP-2 levels (F (8, 18) = 6.606, *p* < 0.001; Figure 8A). Post hoc Tukey’s test indicated that both FXT and DZP significantly increased MMP-2 activity compared with the NS group (*p* < 0.01). Similarly, AST administration at all tested doses enhanced MMP-2 activity, with the most pronounced effect observed at 10 mg/kg (*p* < 0.001 vs. NS).

To further explore the involvement of serotonergic, opioid, and muscarinic pathways in the AST-induced effects, antagonists were administered prior to AST (10 mg/kg). Co-administration of FLU, ATR, and NAL attenuated the enhancing effect of AST on MMP-2 activity, with the reduction reaching statistical significance particularly in the NAL-pretreated group (*p* < 0.01).

In contrast, analysis of MMP-9 activity showed no significant differences among experimental groups (F (8, 18) = 1.33, *p* = 0.29; Figure 8B).

## 4 Discussion

In this study, administration of AST at 5, 10, and 15 mg/kg significantly decreased key anxiolytic-like and depressive-like behaviors in rats, with effects comparable to those of well-established anxiolytic and antidepressant drugs such as DZP and FXT. The behavioral benefits of AST were notably reversed by receptor antagonists FLU, ATR, and NAL.

Importantly, the behavioral improvements were accompanied by significant biochemical changes. Astaxanthin markedly increased serum antioxidant markers, including catalase and reduced GSH, while simultaneously decreasing nitrite levels, suggesting a reduction in oxidative and nitrosative stress. Furthermore, AST modulated inflammatory MMPs, significantly increasing MMP-2 activity and relatively decreasing MMP-9 activity, which may contribute to neuroplasticity and anti-inflammatory processes associated with improved mood and reduced anxiety. The modulation of these biochemical parameters was also attenuated by FLU, ATR, and NAL, reinforcing the role of these neurotransmitter systems in mediating both the behavioral and biochemical effects of AST.

The increasing prevalence of anxiety and depression underscores the urgent need for effective therapeutic interventions. Conventional pharmacological approaches, such as SSRIs and benzodiazepines, while effective in many cases, often come with delayed onset, side effects, and risks of dependence (Dunlop and Davis, 2008; Edinoff et al., 2021). In this context, AST, a natural carotenoid primarily derived from algae and seafood, known for its potent antioxidant properties (Fakhri et al., 2018), emerges as a promising candidate for alleviating anxiety and depression.

The findings of our study indicated that AST administration significantly reduced anxiety-like behaviors in rats, as evidenced by behavioral tests including the open field test, light-dark box test, and elevated plus maze. In all these tests, a dose of 10 mg/kg of AST proved to be the most effective. The fact that the 10 mg/kg dose was the most effective suggests a dose-dependent relationship, in which higher doses may not necessarily yield greater effects and could instead lead to diminished efficacy or potential side effects (Cook and Karim, 2013). In the open-field test, rats treated with AST showed significant reductions in crossing, rearing, and grooming. Although these changes may suggest an anxiolytic-like effect, decreased locomotor and rearing activity can also reflect reduced general activity or mild motor suppression rather than enhanced exploratory behavior. Therefore, the open field findings were considered alongside the results of the elevated plus maze and light-dark box, which more specifically assess anxiety-related responses. Furthermore, following pretreatment with AST, the rats showed a greater tendency to remain in the light areas during both the light-dark box and elevated plus maze tests, suggesting a decrease in anxiety-like behavior. In earlier research, Nishioka et al. found that mice spent more time in the open/light arm after receiving oral doses of AST (Nishioka et al., 2011).

Moreover, the antidepressant-like effects of AST were demonstrated through reduced immobility times in the tail suspension and forced swimming tests. Immobility in these tests is commonly interpreted as a behavioral marker of despair, resembling symptoms of depression in humans (Krishnan and Nestler, 2011). The reduction in immobility times among rats treated with AST suggests a potential enhancement of mood and reduction in depressive-like states, supporting the notion that AST could represent a viable alternative to traditional antidepressants.

Recent research suggests that AST may possess antidepressant effects and could potentially improve sleep quality, which is often impaired in individuals with depression (Peng et al., 2023). Oxidative stress and neuroinflammation are well-recognized contributors to the pathophysiology of depression and anxiety disorders (Nady et al., 2024). Elevated levels of oxidative markers such as reactive oxygen species (ROS), nitrites, and pro-inflammatory cytokines have been reported in both clinical and preclinical studies of these psychiatric conditions, leading to neuronal damage and altered neurotransmission (Hovatta et al., 2010). Beyond behavioral outcomes, we demonstrated that AST significantly modulates key biochemical markers associated with oxidative stress and neuroinflammation, a finding consistent with previous studies. Astaxanthin has been shown to reduce oxidative stress in the brain, which is linked to mood disorders. It can lower levels of pro-inflammatory markers and promote neurogenesis by crossing the blood-brain barrier (Jiang et al., 2016; Rasmus and Kozłowska, 2023). Neurochemical assays indicate that *trans*-AST increases serotonin levels in key brain regions, including the hippocampus and frontal cortex, which are critical for mood regulation (Jiang et al., 2017). Rastinpour et al. (2025) showed that AST increased catalase and GSH levels while decreasing nitrite and NF-κB expression in a rat model of neurodegeneration and cognitive impairment (Rastinpour et al., 2025). Similarly, AST reduced oxidative stress markers and inflammatory cytokines following treatment in spinal cord injury models, thereby contributing to neuroprotection and functional recovery (Masoudi et al., 2021). In addition, AST has been shown to reduce MMP-9 expression and activity in rat brains, associated with improvements in blood-brain barrier integrity and neurological function (Zhang et al., 2015). Astaxanthin has been demonstrated to prevent depression-like behaviors in diabetic mice by inhibiting neuroinflammation in key brain regions such as the hippocampus (Zhou et al., 2017). We previously showed the critical roles of MMP-2 and MMP-9 in neurological diseases, including spinal cord injury (Bahmani et al., 2025; Moradi et al., 2025) and Alzheimer’s disease (Fakhri et al., 2025; Rastinpour et al., 2025). In such disorders, while MMP-2 plays neuroprotective roles, MMP-9 is overexpressed and plays inflammatory and neurodegenerative roles. Additional reports also highlighted the links between MMP-2, synaptic remodeling or neuroplasticity (Huntley, 2012; Wiera and Mozrzymas, 2021).

The mechanistic investigations into AST’s effects revealed interactions with multiple neurotransmitter systems instrumental in regulating mood and anxiety. Specifically, the involvement of the GABAergic system was highlighted using FLU, a GABA-A receptor antagonist, which significantly reversed AST’s anxiolytic effects. This suggests that AST may enhance GABAergic signaling, potentially increasing GABA availability or receptor sensitivity, thereby leading to anxiolytic effects. The GABA-A receptor, particularly its α1βγ2 subtype, plays a pivotal role in mediating the calming effects of GABA, and alterations in its signaling can profoundly affect anxiety and mood disorders (Luscher et al., 2011). Research indicates that alterations in GABA-A receptor signaling can significantly influence anxiety levels. For instance, increased activity at GABA-A receptors has been linked to reduced anxiety behaviors in animal models, particularly within the amygdala, a brain region critical for emotional regulation (Gauthier and Nuss, 2015). Conversely, deficits in GABAergic transmission have been associated with heightened anxiety and mood disorders. Studies show that individuals with anxiety disorders often exhibit changes in GABA-A receptor expression and function (Luscher et al., 2011). In behavioral despair paradigms such as the forced swim test, reductions in immobility time may reflect not only antidepressant activity but also decreased anxiety, stress-induced behavioral inhibition, or altered coping strategies. Through potentiation of GABA-A receptor signaling, DZP can reduce anxiety-related behavioral suppression, which may secondarily manifest as an antidepressant-like behavioral profile without exerting genuine antidepressant efficacy (Petty et al., 1995).

Further supporting the role of GABAergic mechanisms, our findings also indicated interactions with cholinergic signaling, as evidenced by the effects of ATR, a muscarinic receptor antagonist.

Research indicates that dysfunction in cholinergic signaling, especially in the habenula, is associated with depression-like behaviors. In animal models, downregulation of cholinergic genes has been observed in response to chronic stress, leading to symptoms such as anhedonia (loss of pleasure) and mood despair. For instance, knocking down the enzyme choline acetyltransferase (CHAT) in the habenula induced anhedonia-like behavior that was resistant to conventional antidepressants like FXT (Han et al., 2017). The cholinergic-adrenergic hypothesis posits that a high cholinergic tone relative to adrenergic signaling promotes depressive symptoms (Dulawa and Janowsky, 2019). This theory has been supported by various studies showing that administration of acetylcholinesterase inhibitors (AChEIs) can induce depressive symptoms in both humans and animal models. For example, physostigmine, an AChEI, has been shown to increase immobility in forced swim tests, a standard measure of depressive behavior (Fitzgerald et al., 2020). Mineur et al. reported that a decrease in hippocampal AChE activity led to increased anxiety and depression-like behaviors (Mineur et al., 2013). Interestingly, antimuscarinic agents like scopolamine have demonstrated rapid antidepressant effects in clinical settings. These findings suggest the cholinergic system as a potential target for new antidepressant treatments (Navarria et al., 2015).

Additionally, in our study, the opioid system’s role in AST’s mechanism of action was underscored by the use of NAL, an opioid receptor antagonist, which significantly diminished the anxiolytic and antidepressant effects of AST. The opioid system plays a crucial role in mood regulation and is significantly involved in the pathophysiology of mood disorders such as depression and anxiety (Colasanti et al., 2011; Jelen et al., 2022). Research indicates that the endogenous opioid system is often dysregulated in individuals with MDD. This dysregulation can lead to altered stress responses, anxiety, and impaired social bonding, which are core features of depressive symptomatology (Peciña et al., 2019). Specifically, MDD is characterized by symptoms such as sleep disturbances, anhedonia, depressed mood, anxiety, and cognitive difficulties. Many of these functions are influenced by the μ-opioid receptor system (Nummenmaa et al., 2020). The opioid system interacts with several other neurotransmitter systems involved in mood regulation, including serotonin and dopamine pathways. This crosstalk is essential for maintaining emotional balance and responding adaptively to stress. Dysregulation within this system can lead to heightened vulnerability to stress and the development of anxiety disorders alongside depression (Higginbotham et al., 2022).

Given limitations, acute dosing does not model chronic mood disorders; employing chronic models will be more helpful. Evaluating the brain activities of MMPs, sex differences in the antidepressant and anxiolytic effects of AST, and the distinction between behavioral phenotype improvement and disease modification are acknowledged.

## 5 Conclusion

This study demonstrated that AST significantly and dose-dependently improved anxiety- and depression-like behaviors in rats by increasing serum antioxidant levels, decreasing nitrite levels, and regulating MMPs. Such effects were modulated by GABAergic, cholinergic, and opioid neurotransmitter systems. The findings highlight the potential of AST as an adjunct or alternative therapy for mood disorders, targeting oxidative stress, neuroinflammation, and neurotransmitter signaling pathways.

Future studies should further explore the translational potential, sex differences, optimal dosing, and long-term safety profile of AST to establish it as a viable intervention in clinical settings.

## Data Availability

The original contributions presented in the study are included in the article/supplementary material, further inquiries can be directed to the corresponding authors.
